# Population Genomics of the Euryhaline Teleost *Poecilia latipinna*

**DOI:** 10.1371/journal.pone.0137077

**Published:** 2015-09-03

**Authors:** J. C. B. Nunez, T. P. Seale, M. A. Fraser, T. L. Burton, T. N. Fortson, D. Hoover, J. Travis, M. F. Oleksiak, D. L. Crawford

**Affiliations:** 1 University of Miami, Rosenstiel School of Marine and Atmospheric Science, 4600 Rickenbacker Causeway, Miami, FL 33149, United States of America; 2 Department of Biology, Florida State University, Tallahassee, FL 32306, United States of America; National & Kapodistrian University of Athens, Faculty of Biology, GREECE

## Abstract

Global climate change and increases in sea levels will affect coastal marine communities. The conservation of these ecologically important areas will be a challenge because of their wide geographic distribution, ecological diversity and species richness. To address this problem, we need to better understand how the genetic variation of the species in these communities is distributed within local populations, among populations and between distant regions. In this study we apply genotyping by sequencing (GBS) and examine 955 SNPs to determine Sailfin molly (*Poecilia latipinna*) genetic diversity among three geographically close mangrove salt marsh flats in the Florida Keys compared to populations in southern and northern Florida. The questions we are asking are whether there is sufficient genetic variation among isolated estuarine fish within populations and whether there are significant divergences among populations. Additionally, we want to know if GBS approaches agree with previous studies using more traditional molecular approaches. We are able to identify large genetic diversity within each saltmarsh community (π ≈ 36%). Additionally, among the Florida Key populations and the mainland or between southern and northern Florida regions, there are significant differences in allele frequencies seen in population structure and evolutionary relationships among individuals. Surprisingly, even though the cumulative F_ST_ value using all 955 SNPs within the three Florida Key populations is small, there are 29 loci with significant F_ST_ values, and 11 of these were outliers suggestive of adaptive divergence. These data suggest that among the salt marsh flats surveyed here, there is significant genetic diversity within each population and small but significant differences among populations. Much of the genetic variation within and among populations found here with GBS is very similar to previous studies using allozymes and microsatellites. However, the meaningful difference between GBS and these previous measures of genetic diversity is the number of loci examined, which allows more precise delineations of population structure as well as facilitates identifying loci with excessive F_ST_ values that could indicate adaptive divergence.

## Introduction

Global climate change (GCC) is a driving force behind many disturbances affecting global ecosystems [1, 2]. Among the ecosystems disturbed by climate change, coastal habitats are particularly vulnerable. They are threatened not only by increasing temperatures and rising sea levels [3], but also by anthropogenic stressors such as pollution, overharvesting, and habitat alteration [4, 5].

Several studies have shown that ecosystems displaying broader ranges of biodiversity or species richness tend to be more resistant or resilient to disturbances [6–9]. More recent studies have shown that genotypic diversity plays an analogous role to species diversity, *i*.*e*. genetically diverse populations are more resilient in the face of disturbances than those that are less diverse [5, 10–12]. As a result, the *International Union for Conservation of Nature* (IUCN) has declared the conservation of genetic diversity to be an necessity [13]. Genetic diversity may also be important to enhance the ability of populations to adapt to rapid environmental changes [14–16]. Thus for conservation practices, especially with GCC, we need to understand not only the spatial distribution of population size and density but also the distribution of genetic diversity.

South Florida Keys coastal habitats may be particularly susceptible to GCC. These ecosystems have been under constant threats of habitat loss and reduction in species diversity due to rising sea levels [17, 18]. In the Florida Keys, these coastal habitats, predominantly mangrove-dominated salt marshes, are subdivided due to human development and the island habitat. Furthermore, the populations in these mangrove-dominated salt marshes can be isolated from one another because they are not flooded by daily tides that could allow easy movement of individuals among locations but instead are only occasionally flooded by seasonal high tides, hurricanes, and rainfall [18, 19]. Characterizing the genetic diversity within and among these coastal habitats can help us ascertain a) if individual populations harbor substantial genetic variation, b) whether the distribution of genetic variation among populations suggests that individual populations have some connections *via* the exchange of migrants, and c) whether there is evidence that populations occupying even slightly different habitats may show adaptive differentiation. Addressing these issues provide the necessary data to help inform conservation efforts.

In this study we focus on populations of the euryhaline fish, sailfin mollies (*Poecilia latipinna*) inhabiting mangrove saltwater flats. Sailfin mollies are a well-adapted species of topminnow with extraordinary resilience to salty, fresh and brackish waters that inhabit Florida saltwater flats. We apply Genotyping by Sequencing (GBS) to define the variation at 955 single nucleotide polymorphic sites (SNP). We use these data to address two broad questions: i) do GBS approaches agree with previous studies using more traditional molecular approaches and ii) what is the level of genetic variation within and among populations. Similar to previous studies using allozymes and microsatellites [20, 21], the GBS data suggest that most of the variation in southern Florida populations is within populations. Additionally, the large number of loci allows us to examine whether the distribution of some SNPs suggests adaptive divergence among populations. We conclude that even in the Florida Keys (<10 km apart) there is readily detectable genetic divergence among these well-connected populations.

## Materials and Methods

**Fig 1:**
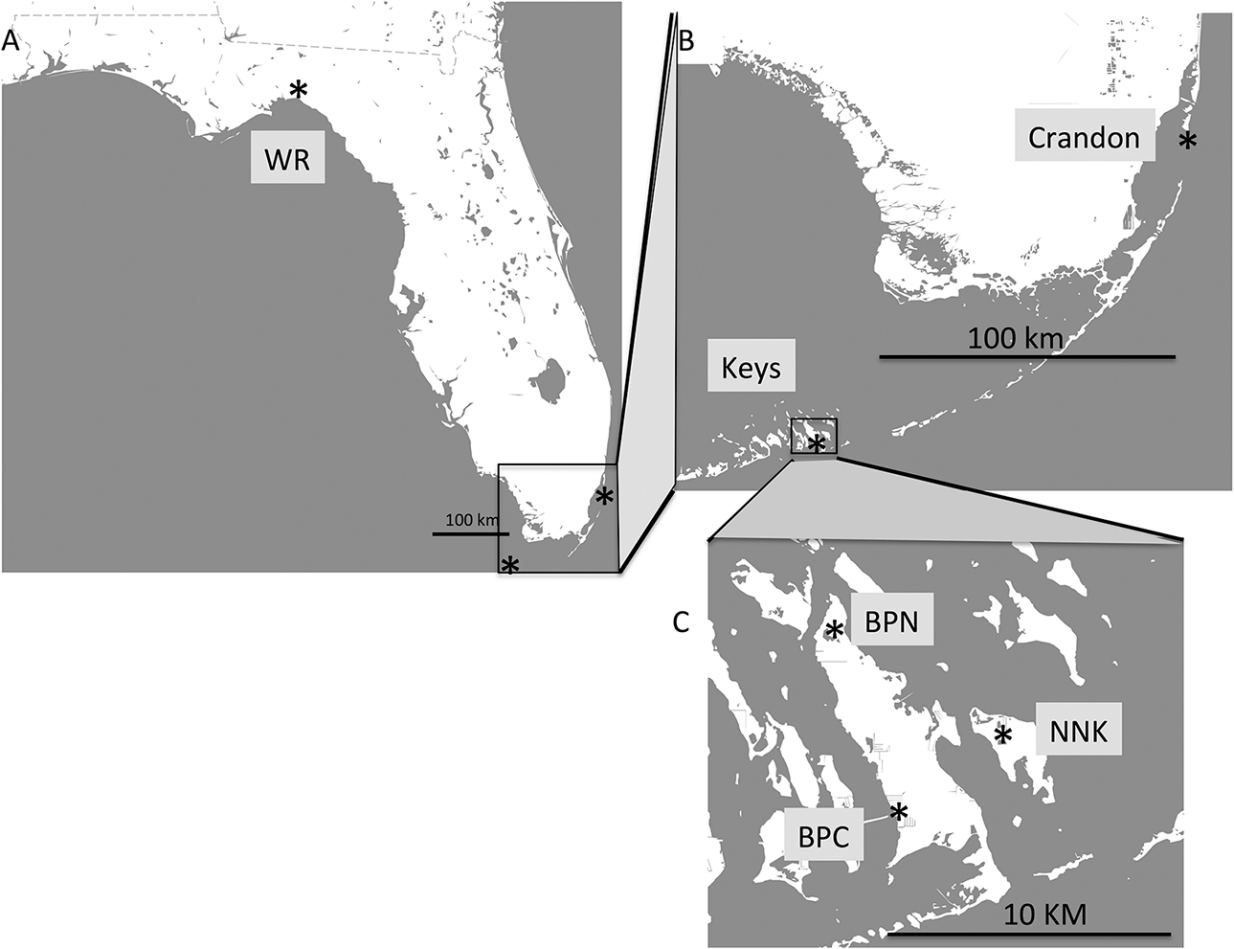
*Poecilia latipinna* collection sites. The study uses a nested analysis for individuals collection across Florida in two two regions, **A:** North Florida and South Florida. Within the southern regions were two locations, **B:** Crandon and the Florida Keys. Within the Florida Keys, individuals were collected at three locations, **C:** Big Pine Key N, Big Pine Key C and No Name Key.

Sailfin mollies were collected from five populations in the spring of 2014 (Table 1). These five populations were from 2 regions: North and South Florida (Fig 1A). Within South Florida, fish were isolated from two locations: Crandon Park in Miami-Dade County and the Florida Keys (Fig 1A). Within the Florida Keys are three populations: Big Pine Key–No Name Rd. (BPN), Big Pine Key–Cahill Rd. (BPC), and No Name Key (NNK; Fig 1B). All South Florida populations were collected from spatially isolated ponds with different environmental conditions (Table 1). One hundred and twenty individuals (thirty *per* site) were collected from South Florida populations; sixteen individuals were collected from the North Florida population. Fish were collected near shore using minnow traps and dip nets. All fish were returned to the local environment after removing small (<10mm^2^) fin clips. Collected fish were measured for standard length, and fin clips were stored in 320 ul of Chaos buffer (4.5M guanadinium thiocynate, 2% N-lauroylsarcosine, 50mM EDTA, 25mM Tris-HCL pH 7.5, 0.2% antifoam, 0.1M ß-mercaptoethanol) and stored at 4°C prior to processing. Genomic DNA was isolated using a silica column [22]. Genomic DNA quality was assessed *via* gel electrophoresis, and concentrations were quantified using Biotium AccuBlue™ Broad Range dsDNA Quantitative Solution according to the manufacturer’s instructions.

**Table 1:** Collection Sites and Sample Size.

Location	N (120 individuals)	Latitude	Longitude	Salinity (‰)
North Florida, Wacissa River (WR)	14	30°20’21.29”N	83° 59’32.48”	Fresh
Miami-Dade, Crandon Park	24	25°43'31.17"N	80° 9'4.09"W	35.0
Big Pine Key, Cahill Rd (BPC)	27	24°40'6.92"N	81°22'8.44"W	50.0
Big Pine Key, No Name Road (BPN)	27	24°43'39.08"N	81°23'44.45"W	45.0
No Name Key (NNK)	28	24°42'5.95"N	81°19'52.65"W	35.0

N sample size after filtering data (see text), *Salinity was determine when collecting samples.

GBS was preformed as described [23] using the restriction enzyme *Ase I*. Adaptors (0.4 pmol/sample) were ligated to 50 ng of gDNA. The GBS library was sequenced on a single lane using Illumina HiSeq 2500 with a 100 bp single end read (Elim Biopharmaceuticals, Inc.). The UNEAK GBS analysis pipeline, TASSEL [24], was used to call SNPs using Bowtie. Only the first 64 bp of each sequence (Tag) was retained. This minimizes the errors in sequence calls associated with the end of 100 bp sequences [24].

We examined pairwise differences within and among populations (number of differences between a pair of individuals per SNP*100%), heterozygosity, Hardy-Weinberg equilibrium, migration rates, F_ST_ values, and outlier F_ST_ identification using the “ape” package in the R statistical computing environment [25], Arlequin v.3.5.12 [26] and LOSITAN [27]. F_ST_ values calculated by FDist2 LOSITAN [27] and Arlequin v.3.5.12 [26] were very similar, but the p-values were different because they are based on different permutations of the data. FDist2 defines significance based on permutation of SNPs with similar expected heterozygosites. Arlequin defines significance permutations of individuals. Migration estimations were determined using a matrix of Slatkin’s linearized F_ST_ [28]. Estimates of effective population size (Ne) use Arlequin v.3.5.12 [26] and a Bayesian historic approximation [29] using > 250,000 simulations. Population structure was defined by fastSTRUCTURE [30], and RaXML was used to build a maximum likelihood tree [31]. Isolation by distance (IBD) and discrimination analyses used the R-package “adegenet” [32, 33]. IBD was tested using a Mantel test between a matrix of genetic distances and a matrix of geographic distances. Discriminant analysis of principal components (DAPC) was used to discern differences among populations. In contrast to Structure, DAPC does not assume unrelatedness, and therefore, potentially closely related individuals can be included in the analysis.

Analyses for outlier SNPs were performed using LOSITAN (FDist2) [34] and Arlequin v.3.5.12. FDist2 comparisons were performed among the five populations, among the three Florida Keys populations and the Miami-Dade population, among the three Florida Keys populations and northern *versus* southern populations. For the last FDist2 comparison (North *vs*. South), the four South Florida populations were treated as a single population. Comparisons were run using 500,000 simulations at a 99% confidence interval and a false discovery rate (FDR) of 0.01. Arlequin outlier analyses were conducted using a hierarchical model [35] with 10,000 coalescent simulations and 100 demes simulated per group. Coalescent simulations between the three Florida Keys could not be performed individually due to their high migration rate. Thus, we treated these locations as a single population in the hierarchical outlier test. We performed three comparisons: 1) among all populations (Keys (as a single population), Crandon Park in Miami-Dade and North Florida), 2) between the Keys and Crandon Park in Miami-Dade, and 3) between South Florida (treating the four South Florida locations as a single population) and North Florida populations.

To identify the genes with outlier SNPs, we searched GenBank at the National Center for Biotechnology Information (NCBI) using the 64 bp sequence tags containing these SNPs. Specifically, the nucleotide Basic Local Alignment Search Tool (nBLAST) at NCBI was searched among Actinopterygi sequences (ray-finned fishes; taxid:7898), using highly similar sequences search algorithm (megaBLAST) and accepting matches with E values less than 1oE-5. All other parameters were used as default.

Ethics Statement: Fieldwork was completed within publically available lands and no permission was required for access. *Poecilia latipinna* is not endangered or protected status, and small marine minnows do not require collecting permits for non-commercial purposes. All fish were captured in minnow traps with little stress and released in less than 1 hour. Fish were returned to the site of capture with little obvious effect of sampling small fin clips (< 4mm x 4mm). IACUC (Florida State University Animal Care and Use Committee) approved procedures were used for non-surgical tissue sampling with captures and release.

## Results

### Nested Sampling

There is a nested set of five Florida populations with a total of 120 individuals (Fig 1, Table 1). The nesting consists of the northern and southern region: the southern region (Crandon Park in Miami-Dade County and the Florida Keys) and the northern region (WR, n = 14) in the Florida panhandle. Within the southern region were two locations: the Florida Keys with three populations (Fig 1B; Big Pine Key: BPC, n = 27, BPN, n = 27 and No Name Key: NNK, n = 28, and Crandon Park in Miami-Dade County, n = 24). The geographic distances among the three populations in the Florida Keys are small (< 10 km). Within the southern regions, Crandon Park is approximately 180 km from the Keys, and between the southern and northern regions, the distance is > 600 km (Fig 1). The four populations in the south Florida region were collected in mangrove salt flats with salinities ranging from 35 ppt to 5o ppt (Table 1). The most northern population was collected in freshwater at the headwaters of the Wacissa River.

Population sizes were estimated using a Bayesian approach [29]. These data suggest that population sizes were between 10,000 to approximately 90,000 in southern Florida but were approximately in the 100–1,000 range for the single northern population. These data are similar to those estimated in the hierarchical AMOVA assuming mutation rates of 1E-9.

### Genomic DNA and Read Depth

*Poecilia latipinna* genomic DNAs (gDNA) were used to create bar-coded, reduced representation, genomic libraries [23]. Each SNP is found on a single non-overlapping “tag” (64 bp sequence) where the sequence is initiated at the restriction site. An initial total of 740,993 SNPs were covered by 128 million 64 bp reads (tags). Of these, 18,255 SNPs had a minimum of five reads per SNP and 5% minimum allele frequencies across all samples. We removed 24 of 144 individuals with <10% calls and removed SNPs not called in 80% of individuals, resulting in 1,320 SNPs in 120 individuals (Table 1). Furthermore, we removed SNPs in which observed heterozygosity significantly exceeded expected heterozygosity (HWE, p < 0.01) and SNPs with significant linkage-disequilibrium (p < 0.01, or with r^2^ > 0.2). This resulted in 955 SNPs with 6.48 millions reads. The average number of reads per SNP was 6,787 (range: 775 to 16,468). Excluding individuals with no reads (*i*.*e*., there were no sequences for a SNP), the average number of reads per SNP per individual was 64 (range: 8–137). Individuals with no reads are excluded because they do not contribute to measures of allele frequencies, or population genetic measures.

### SNP Loci

Table 2 provides the average frequency of the major allele and average expected heterozygosity. The major SNP allele is defined by the allele with a frequency of >0.5 across all five populations. The average major allele frequency across all five populations for 955 SNPs was 81% with 95% within 8% of this value (Table 2A). Within each of the five populations, the average major allele frequency ranged from 70% for WR to 82% for BPC. Across all five populations, the average heterozygosity was 27.6% with 95% of loci within 8% of this value. Within each of the five populations, heterozygosity ranged from 14% to 24% (Table 2B).

**Table 2:** A: Major Allele frequencies and B: Heterozygosity for 955 SNPs.

A:							
Major Allele Frequencies		Overall	BPC	BPN	NNK	Crandon	WR
	Avg	0.810	0.827	0.825	0.825	0.818	0.700
	95%-	0.802	0.817	0.815	0.815	0.806	0.676
	95%+	0.818	0.837	0.835	0.835	0.829	0.724
	MIN	0.500	0.227	0.220	0.260	0.000	0.000
	Max	0.950	1.000	1.000	1.000	1.000	1.000
B:							
Heterozygosity		Overall	BPC	BPN	NNK	Crandon	WR
	Avg	0.276	0.238	0.240	0.238	0.233	0.142
	95%-	0.268	0.227	0.229	0.227	0.222	0.131
	95%+	0.284	0.248	0.250	0.248	0.244	0.154
	MIN	0.095	0.000	0.000	0.000	0.000	0.000
	Max	0.500	0.500	0.500	0.500	0.500	0.500

Major alleles are alleles with > 0.5 frequency across all populations

Among populations, there were 30 fixed differences between the northern FL and southern FL populations (fixed difference in allele frequency = 100% *versus* 0%; Table 3), and no other contrast between populations presented any fixed differences. The average difference in allele frequency between the northern and southern regions is 34%. Within the southern region, there are no fixed differences and an average of 17% difference in allele frequencies. Within the Florida Keys there is an average of 10% difference in allele frequencies.

**Table 3:** Differences in Allele Frequencies for Major Allele.

	w/in FL Keys	w/in S. FL	South FL *vs*. North FL
Number of Fixed differences	0	0	30
Average difference in frequencies for the major allele across all 5 populations	0.099	0.169	0.335
+95%	0.095	0.161	0.353
-95%	0.104	0.176	0.317

Differences for “w/in FL Keys” are among the Key populations with the largest *versus* smallest allele frequency.

Differences for “w/in S.Fl” are among the Southern populations (Keys & Crandon) with the largest *versus* smallest major allele frequency.

Differences for “South FL *vs*. North FL” are average of major South–North allele frequencies

### Genetic Diversity within Populations

Within all four southern Florida populations (Crandon and Keys) the pairwise differences (π) among individuals is substantial and similar to π among all individuals (i.e., among all 120 individuals ignoring populations, Table 4). In the most northern freshwater population (WR), π is o.228 or >30% lower than π within each population in the southern region (o.228 compared to an average of 0.355). This reduction in π for the WR population is also reflected in the number of monomorphic sites: WR has approximately 4-fold more monomorphic sites than the southern populations. The frequency of homozygotes could arise from too few reads per SNP and thus could inflate the frequency of invariable or monomorphic SNPs in a population. To avoid this potential source of bias, the number of monomorphic sites was calculated only if there were ten or more individuals with ten or more reads per SNP per individual. Thus, the greater number of monomorphic sites in the northern FL population is not due to low sequence depth.

**Table 4:** Pairwise Differences.

	Avg.	Min.	Max.	Mono
All	0.393	0.187	0.630	0
BPC	0.360	0.264	0.485	99
BPN	0.357	0.269	0.477	99
NNK	0.357	0.279	0.458	105
Crandon	0.348	0.277	0.407	130
WR	0.228	0.187	0.260	472

Mono: monomorphic, number of invariable SNPs with at least 10 individuals with a minimum of 10 reads per individual per SNP

### Genetic Diversity Among Populations

To examine the diversity among populations, we applied an analysis of molecular variance (AMOVA, [36]) among 3 groups: northern Florida (WR), Crandon Park and the Florida Keys. Using all 955 SNPs indicates there is significant difference among all three groups (p <0.0001). Much (71%) of the variation is within populations, and 28% is among groups (Keys, northern or southern Florida). Considering only the four Southern populations (Crandon and the three Keys), 92% of the variation is within populations and 8% is among populations. Examining only the Florida Keys, 99% of the variation is within populations, with only 1% of the genetic variation among the three Florida Keys populations. These divergence patterns are reflected in the overall F_ST_ value using all 955 SNPs (Table 5).

**Table 5:** Overall FST Values Between Population with P-values.

Populations		P-values				
		BPC	BPN	Crandon	WR	NNK
F_ST_ values	BCP		< 0.0001	< 0.0001	< 0.0001	< 0.0001
	BPN	0.01079		< 0.0001	< 0.0001	< 0.0001
	Crandon	0.083	0.08176		< 0.0001	< 0.0001
	WR	0.46237	0.45967	0.44963		< 0.0001
	NNK	0.01212	0.01131	0.07919	0.4547	

P-values calculated after 10,100 replications

**Fig 2:**
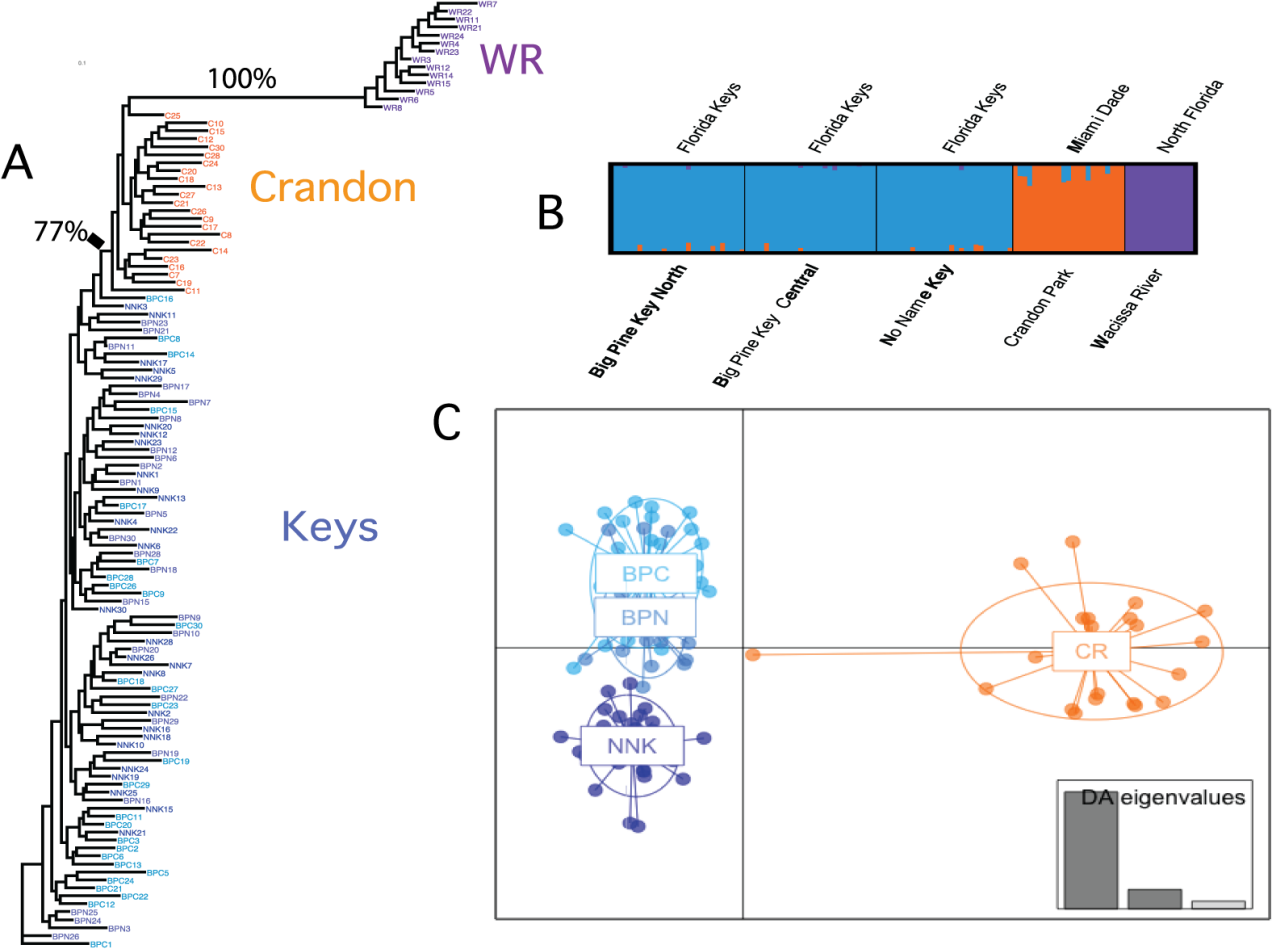
Population divergence. Maximum likelihood tree, structure plot and DAPC plot. **A:** Maximum likelihood tree of all 120 individuals. The three Florida Key populations are in green, light blue and dark blue, Crandon Park is orange and north Florida (WR) is purple. The only two branches with bootstrap values > 50% are labeled with frequency of support. **B:** Structure plot from the most likely K = 3. **C:** DAPC plot for the four south Florida populations: Big Pine N is blue Big Pine C is light blue, No Name Key is deep blue and Crandon Park is orange. Insert is the percent explained variance for the first three discrimination eigenvalues.

The overall F_ST_ values show a divergence pattern where WR, the most northern population, has the largest F_ST_ values (~0.45) when contrasted with any of the southern populations. Between southern Florida and the Florida Keys, the F_ST_ value is approximately 0.08, and within the Florida Keys the F_ST_ values are near 0.01. There are three additional sets of results that use the variation in allele frequencies to define divergences among populations (Fig 2): maximum likelihood tree, Structure [37], and discriminant analysis of principal components (DAPC, [38]). The maximum likelihood tree (Fig 2A) uses all 955 SNPs and reveals divergence between the Florida Keys relative to both Crandon Park and WR populations (the only branches supported by bootstraps values > 50%). There is little that distinguishes the three Florida Keys populations, yet Crandon Park and WR form two well-supported clades.

Structure analyses [37] suggest that there are three groups of individuals. Specifically, three groups maximize the marginal likelihood of the entire data set (Fig 2B). The three groups correspond to northern Florida, Crandon and the three Florida Keys populations. To further resolve differences among the four populations in the southern Florida population (Crandon and the three Florida Keys populations), a DAPC analysis [38] was applied to the four southern Florida populations (Fig 2C). DAPC is a multivariate approach that partitions the genetic variation into a between-group and a within-group component using principal components of genetic variation to describe the genetic differences among groups while minimizing the differences within groups. DAPC shows that the first discriminant function separates Crandon from the Keys and the 2^nd^ function separates No Name Key from the two Big Pine Keys populations (Fig 2C). These results reflect the geographic separation of these four populations.

**Fig 3:**
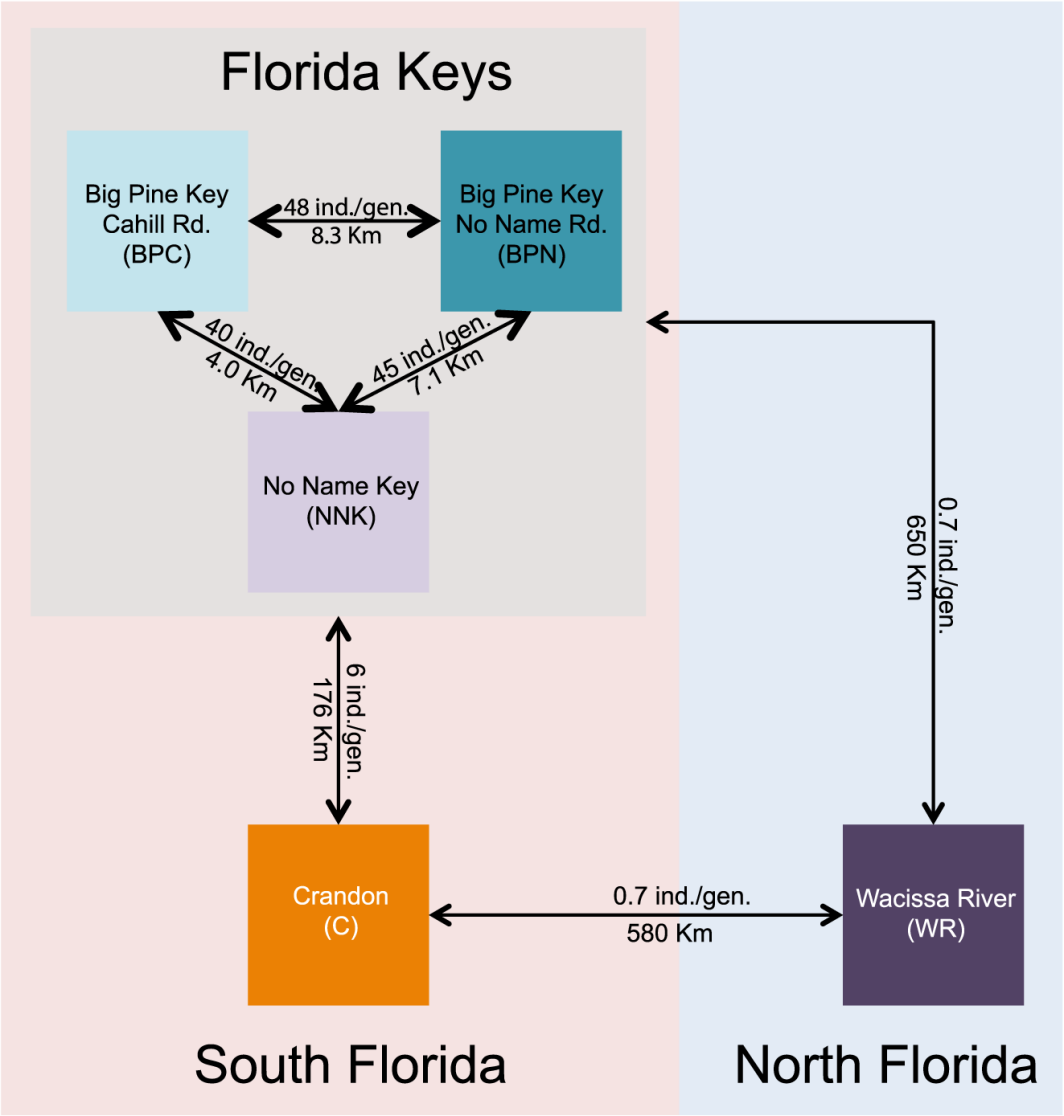
Migration Estimates. Diagram showing the estimated number of migrants *per* generation among all populations. Estimates were determined using a matrix of Slatkin’s linearized F_ST_. Text with the lines between populations shows the estimated migration rate as well as the geographic distance between populations.

The divergence among populations seen in F_ST_ values suggests variation in population connectivity. To estimate this, we calculated migration rate using Slatkin’s distances [28] among these three regions: Northern Florida, Crandon Park and the Keys (Fig 3). The three Keys populations' migration rates are comparatively higher with approximately 44 migrants per generation among all three populations. Between the Keys and Crandon Park there are 6 migrants per generation, and <1 between Southern and Northern Florida (Fig 3).

### Locus-Specific Differences

**Fig 4:**
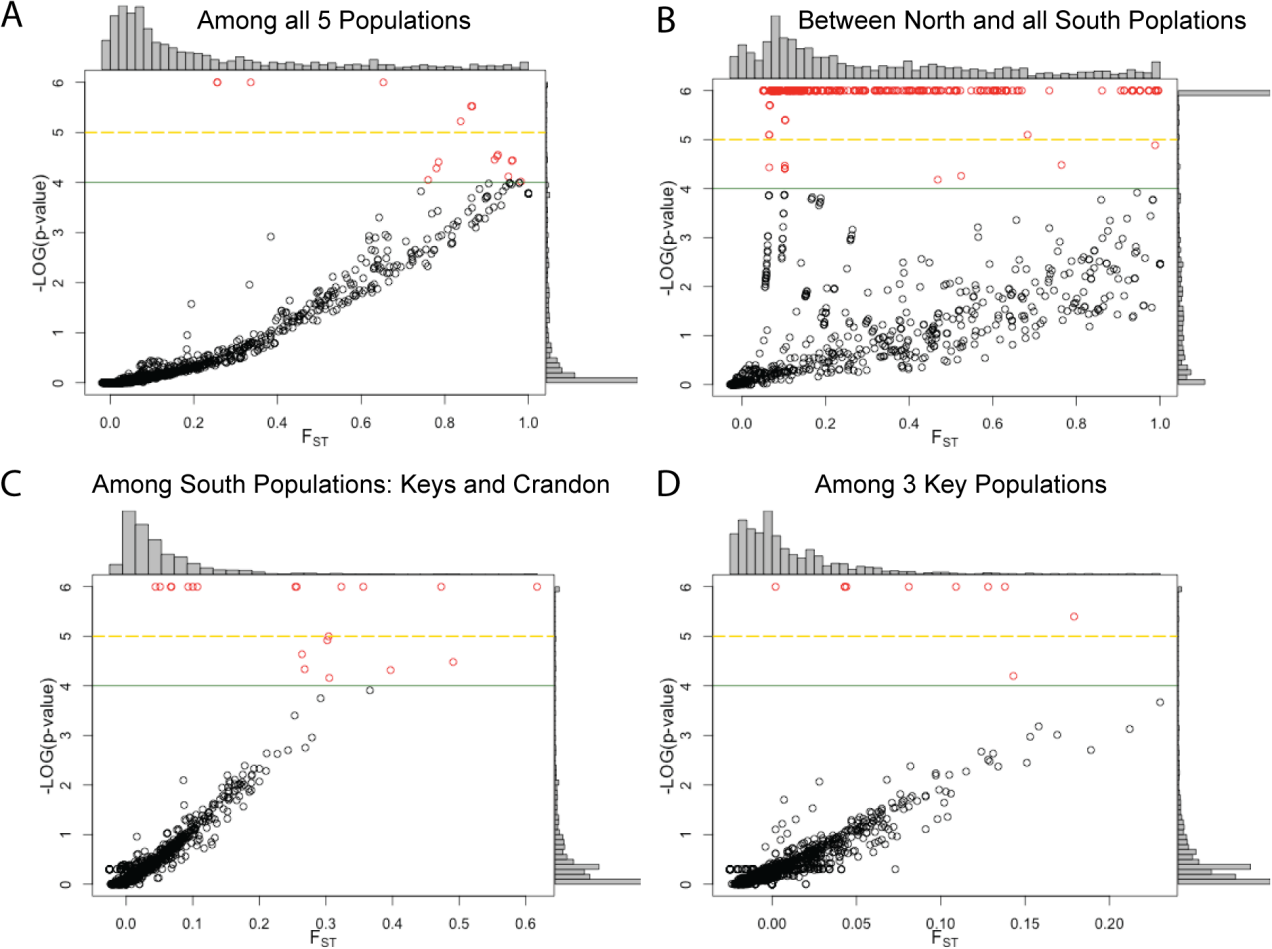
F_ST_ values and p-values for all SNPs. SNPs shown as a function of their F_ST_ values and their corresponding FDR corrected P-value. Histograms show the distribution F_ST_ values (above) and p-values (right) of plot. FDist2 outlier SNPs are shown in Red above the green line. SNPs above the yellow dashed line have p-values below the Bonferroni’s correction threshold (< 10^−5^). Comparisons shown **A:** All 5 populations, **B:** North vs. South Florida (Crandon and the Keys as one population), **C:** South Florida (Crandon *versus* the Keys), **D:** Keys (BPC, BPN, NNK). SNPs with a p-value < 1E-6 were set to 1E-6.

The overall F_ST_ values among populations are significant even though the F_ST_ values are small among the four southern populations (Table 5). However, there are SNPs within the Florida Keys, among the southern Florida populations or among all five populations that have large, significant F_ST_ values (FDist2, p < 0.01; Table 6). The F_ST_ value distribution and associated p-values (as negative log10, *e*.*g*., where 2 = 0.01, 6 = 1e-6) are shown in Fig 4. With 955 SNPs, one would expect ~9 SNPs by chance to have p-values less than 1%. In all cases, the number of SNPs with significant F_ST_ values exceeds this null expectation, dramatically so for the distinction between northern and southern Florida. Furthermore, Bonferroni’s corrected p-values (< 10^−5^) suggest that despite the small geographic distances among the Florida Keys populations, there are SNPs that have significant divergences. We can compare the number of FDist2 significant F_ST_ values (Table 6, Fig 4) with the number found using AMOVA hierarchical analyses [26]. The F_ST_ values for both are nearly identical, but the p-values are different because they are based on different permutations of the data. While the number of significant F_ST_ values is higher for the AMOVA (Table 7), nearly all the significant F_ST_ values (Table 6) were significant with the AMOVA hierarchical analysis: among all populations all 119 (100%) are also significant, and among the population in the southern regions (Crandon vs Keys), 44 out of 48 (92%) share significance. However, for F_ST_ values between the northern and southern regions, only 255 of 481 (53%) have shared significance.

**Table 6:** SNP specific F_ST_ values and FDist2 outliers.

	# SNPs significant (corrected)	Avg. significant F_ST_ value	Min.	Max.	Outliers
5 populations	119 (7)	0.778	0.256	1.000	62
Crandon vs. Keys	48 (14)	0.224	0.044	0.617	23
Keys	29 (9)	0.118	0.02	0.230	11
N vs S	481 (349)	0.338	0.050	1.000	411

Number of SNPs with significant F_ST_ values p < 0.01 (with Bonferroni’s correction, p-value <10^−5^). Average, minimum and maximum F_ST_ values among significant SNP. Outlier loci are based on permutation of data and occur less than 1% with FDR correction of 1% (approximate p-value = 0.003).

**Table 7:** SNPs F_ST_ values in a Hierarchical Island Model test.

	AMOVA Significant SNPs (corrected)	Hierarchial Outliers	Avg. Outlier F_ST_ value	Min. Outlier	Max. Outlier
Among All Populations	514(326)	103	0.886	0.660	1.000
N v S	527(319)	72	0.971	0.916	1.000
Crandon vs. Keys	155(35)	17	0.504	0.393	0.773

Number of SNPs with significant F_ST_ values p < 0.01 (with Bonferroni’s correction, p-value <10^−5^). Average, minimum and maximum F_ST_ values among significant outleier SNP.

To determine whether any of these SNPs may have adaptive significance, an outlier analysis was used to identify SNPs with F_ST_ values that lie outside the 99% distribution with 1% FDR (Table 6, [39]). Results from the FDist2 outlier test found 62 outlier SNPs among all 5 populations (Fig 4A), 23 within South Florida (Fig 4B), 11 among the Keys (Fig 4D), and 411 between northern and southern Florida (Fig 4C).

Among the 411 outlier SNPs, the average number of reads for each SNP among the 120 individuals is 6,705, (range: 1,167 to 12,534). Of these 481 outlier SNPs, 32 had significant nBLAST hits to all bony fish (E-value < 10^−15^). The majority of these (18) were for transcriptional factors or signaling factors (receptors, G-proteins). Among the three Florida Keys populations, which are less than 10 km apart with small overall pairwise genetic distances (F_ST_ values < 0.02), there are eleven outlier SNPs with an average F_ST_ value of 0.104 (range: 0.020–0.230). These 11 SNPs have an average of 4,783 reads (range: 3,3578 to 5,734) among the 71 individuals in the three Florida Keys populations. Only one of these SNPs had meaningful annotation: FAM69A, a cysteine-rich type II transmembrane protein localized to the endoplasmic reticulum.

Outlier tests identify loci with potentially adaptive F_ST_ values that are unlikely to occur because their values exceed the value for all other SNPs with similar heterozygosity [40]. Yet, the false positive rate can exceed 5% depending on the demography of populations and the underlying models used in outlier analysis [40]. FDist2 (Lositan, [27]) uses an island model, and while this seems appropriate for the Keys, we wanted to verify these data using a hierarchical model used in Arlequin [26].

Results from the hierarchical outlier test are shown in Table 7. This model depends on limited migration and thus could not calculate values within the Keys; therefore the three Key locations were treated as one population. These data are similar to the FDist data: among all populations there are >100 SNPs that had outlier F_ST_ values: 72 for the comparison between Northern *versus* Southern regions and 17 in southern Florida (between Crandon and the Keys) comparison.

**Fig 5:**
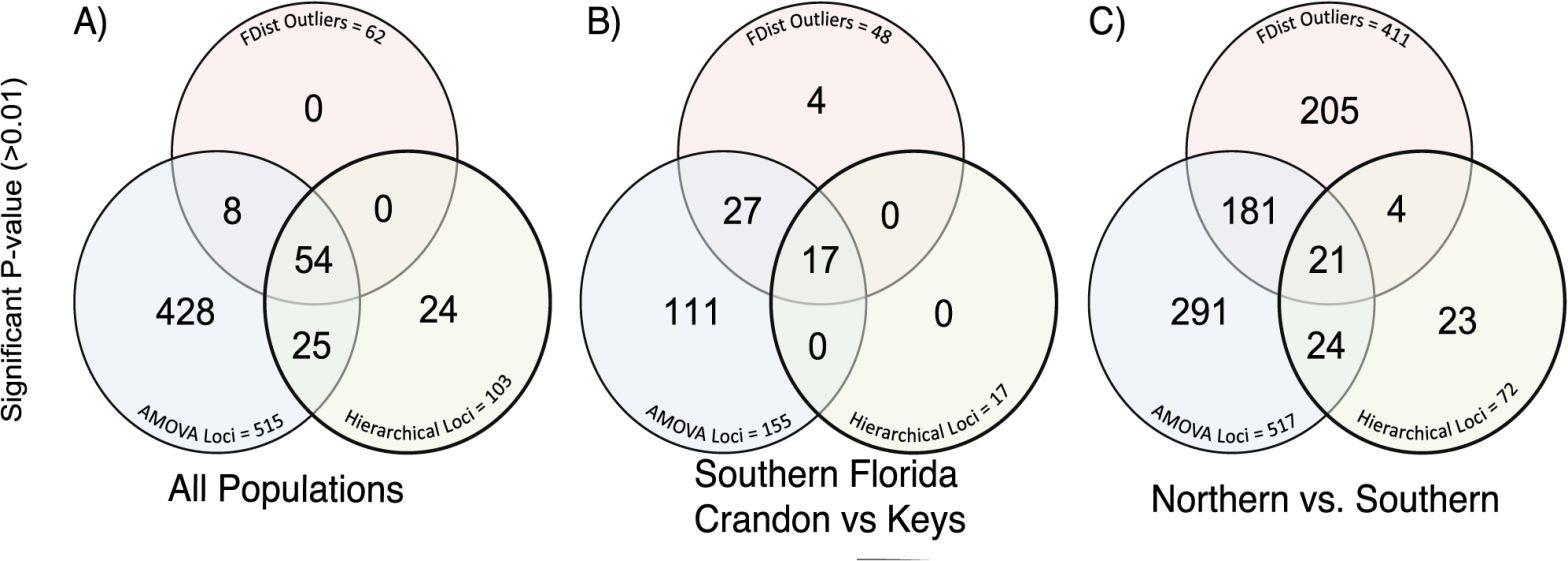
Venn diagrams showing 3-way comparisons of Loci with significant F_ST_ values: FDist outlier test, Hierarchical outlier test, and significant F_ST_ values from the AMOVA: (A) Among all populations: (Keys *vs*. Crandon *vs*. North Florida); (B) Southern Florida (Crandon *vs*. Florida Keys) and (C)North *vs*. South Florida.

In Fig 5 depicts the Venn diagram for shared loci among AMOVA significant F_ST_ values and outlier tests for FDist2 and hierarchical models. For comparison among all populations, 54 out of the 62 (87%) FDist2 outliers are outliers with the hierarchical model. For the comparison within the Southern Region (Crandon *versus* the Keys) 14 of the 23 (61%) FDist2 outlier are outliers with the hierarchical model. Many fewer outliers were shared when comparing the Northern and Southern region (Fig 5): only 20 out of the 411 (5%) FDist2 outliers are outliers with the hierarchical model.

## Discussion

Among coastal habitats, mangrove estuarine forests are changing: they are extending further north with saltwater intrusion associated with sea level rise [3, 18, 41, 42]. How an increase in sea level affects the distribution and size of mangrove estuaries is dependent on human habitation: coastal habitats may be inundated with seawater which could support mangrove habitats, but the presence of human development will preclude mangroves from growing [3, 42, 43]. The movement and potential reduction of mangrove forests by GCC could affect ecological and population dynamics of animals inhabiting these estuaries. A critical factor in the survival of a specific species is the standing genetic variation that allows species to adapt to these changes [14]. To determine the extent of genetic variation and how it is partitioned among populations, we provide data on the genetic diversity in the teleost fish Sailfin molly (*Poecilia latipinna*) by comparing three geographically close Florida Keys populations to a mainland population in south Florida (Crandon Park) and a northern Florida, freshwater population.

Previous studies have demonstrated that *P*. *latipinna* populations have genetic divergence that increases with geographic distance [21], and this diversity is affected by periodic storms or hurricanes [20]. These studies have shown that there is substantial genetic diversity among regions (100s of kilometers apart, *e*.*g*., northern and southern Florida) with little substantial diversity among populations within a region (10s of kilometers apart, [21]. The genetic variation among regions is responsible for significant differences in life history traits (body size, growth and maturity [44–46]). These studies suggest that the changes in allele frequencies across large geographic regions affect important biological traits. Thus, these allozyme and microsatellite data suggest that conservation practices need to consider the preservation of a few local populations among many distant regions. In this study, we examine 955 SNPs loci and demonstrate that even on the smallest geographic scale (< 10 kilometers) there are substantial differences in allele frequencies, which may arise due to natural selection for locally adapted demes.

### SNPs and Population Genetics

For population genetics it is common to examine polymorphic proteins, variable microsatellites or DNA sequences from one or a few genes. Proteins typically have two or few alleles that represent an unknown number of nucleotide changes. Microsatellites have many alleles (approx. 20 in *P*. *latipinna*, [20]) that differ in the number of repeats. Thus, GBS SNP data are most similar to DNA sequences because we are examining nucleotide changes with two alleles at a single position. However, GBS SNP data are different from DNA sequence variation analyses because only variable positions are captured from 64 bp Tags (short sequencing reads trimmed to 64 base pairs). Clearly, examining only polymorphic Tags inflates our measures of nucleotide variation. Yet this is not so different from choosing variable allozymes, which inflates estimates of the amount of protein polymorphism. What we can say is that for polymorphic Tags (Tags with SNPs) there is an average π of 39.3%. To compare GBS SNPs to DNA sequence variation, we can correct for the selection of only polymorphic sites within Tags by dividing by 64 bp/SNP (see below).

### Genetic Diversity within Populations

Genetic diversity was high within the southern populations (three Florida Keys populations and Crandon Park, Miami Dade, Table 4). The genetic diversity is best represented by pairwise differences (π, number of nucleotide differences between pairs of individuals divided by the number of SNPs). The pair-wise differences within each southern Florida population is >34% with a range of 34.8–36.0% (Table 4). These differences among individuals within a population are similar to the value across all individuals in all five populations. That is, the diversity within each of the southern FL populations is similar to the variation among all individuals when ignoring population structure. This similarity of π within and among populations explains the AMOVA results within the Florida Keys where 99% of the variation is within populations. In the northern freshwater population π is substantially less (22.8%).

The second measure of genetic diversity is He (expected heterozygosity), which is approximately 0.23 in the southern populations. In the northern population heterozygosity is much smaller: it is 0.14 or 60% of that found in the southern populations. These data are based on an average of 6,787 reads per SNP (range: 775–16,468), with an average of 57 reads per SNP per individual (range: 6–137). Thus, it is unlikely that this variation is due to sequencing errors because all SNPs have sufficient replication. In comparison to other GBS studies, π values within these sailfin molly populations are similar to those measured in stickleback and natural populations of *Saccharomyces cerevisiae* [47, 48].

These measures of genetic diversity using GBS data are substantial and seem to be much larger than other measures of genetic variation. For example, allozymes in populations from the same region have heterozyosity values of 0.05 [21] with 13% of loci being polymorphic. Yet, the direct comparison between SNPs and allozymes is difficult. Allozyme diversity will underestimate the actual level of genetic variation. For one reason, it is biased because only charged amino acid polymorphisms are discerned in protein gel electrophoresis. For another, proteins represent 100s of amino acids and even detectable allozyme variation may be the aggregation of hundreds of single nucleotide differences [49]. We filtered the SNP data to discover only polymorphic sequences in 64 bp sequences (see above). If one divides the SNP measures of diversity (π and heterozygosity) by 64 (number of nucleotides per variable sequence), π is 0.5% and expected heterozygosity is only 0.004. If we assume only one amino acid substitution among 100 possible amino acids in an allozyme, then 0.13% of amino acids are polymorphic with heterozyosity of 10^−4^ at each amino acid. Although these are crude estimates, they do suggest that GBS provides much more information about polymorphism per locus than allozymes. This is not surprising because neutral divergence is a function of functional constraints [50], and in protein coding genes (*e*.*g*., allozymes) there are fewer nucleotide substitutions that do not have a deleterious effect than in random genomic regions interrogated using GBS. Thus, one expects more variation in GBS studies than when using allozymes. The fact that GBS provides orders of magnitude more loci suggests that these data can reveal more detailed information about the conservation genetics of a species.

We can compare the pairwise individual differences estimated from SNPs, π per bp (0.5%, π/64 bp per sequence), to DNA sequence variation. SNP's π per bp of 0.5% is larger than the π for DNA sequences captured from teleost fish promoters of *Ldh-B* among populations from Georgia to Maine (0.018; Crawford *et al*. 1999) and promoters for CYP1A among populations from New England (0.015; [51]). The values we report here are also larger than the π for DNA sequences estimated in 5 global populations of *Drosophila melanogaster* in the *eve* regulatory elements (0.015; [52] and between *D*. *melanogaster* and *D*. *simulans*, 0.03 [53]. When compared across a wide array of taxa, π = 0.5% is a substantial level of genetic variation relative to most animals [54]. These comparisons to DNA sequence data suggest that, like the comparison to protein polymorphisms, GBS provides a greater variety of genomic regions that have greater polymorphism because there are fewer constraints.

Overall, the data on SNP polymorphism suggest first, that within populations, *P*. *latipinna* has substantial genetic variation in comparison to GBS SNP studies on other outbred species. Additionally, GBS SNP data provide a greater variety of loci that have greater variation than the DNA sequence variation of single genes in many previous studies. Second, the data show that the genetic diversity is higher in the four southern populations than in the single northern population. Third, the data show that in the southern populations, π within populations is similar to π across all individuals. The high frequency of polymorphisms within populations is similar to previous studies on *P*. *latipinna* using allozymes and microsatellites that indicated a large proportion of all genetic variation is found within individual populations [20, 21]. The important point is that while GBS studies examine 100s-1,000s more loci than allozymes or microsatellites, the conclusions about the distribution of variation within and among populations is similar among all three genetic markers and thus is not an artifact of one particular marker.

### Genetic Diversity among Populations

Among the five populations, the four southern Florida populations were sampled in saline or hyper-saline water with little obvious connection to daily tidal flows. These upper-tidal salt flats are inundated at extreme high tides or during storms. These southern Florida populations are geographically separated and environmentally different than the WR freshwater population, and this is clearly seen in the SNP data. WR is clearly distinct from the southern Florida populations: the WR population *versus* southern populations has the largest overall F_ST_ value, the most number of SNPs with significant F_ST_ values, and is the only population with fixed differences in allele frequencies. This divergence between these two distant regions is similar to previous allozyme and microsatellite data [20, 21]. Specifically, based on allozymes, the F_ST_ values among regions including northern Florida and Georgia was 0.21 [21]. We provide a similar comparison by contrasting F_ST_ values for northern *versus* all southern populations. Based on 481 SNPs with significant F_ST_ values, the average F_ST_ value was 0.34.

Without replicates of other freshwater samples or hyper-saline samples in northern Florida, it is difficult to discern what evolutionary factors are affecting the divergence between these two regions. However, the WR population in northern Florida has more monomorphic SNP and lower π and He values than any southern population, suggesting that this population has been historically smaller and more isolated. Both would contribute to greater drift. Additionally, the number of SNPs with significant F_ST_ values is large but highly dependent on the method used to determine significance (Tables 6 and 7). Specifically, only 53% of F_ST_ values share significance between the two approaches used here. This also extends to outlier analyses: most FDist2 outliers are not outliers with hierarchical analysis. These data suggest that demography (bottle neck, complex population structure, etc.) affects the analyses between the northern and southern regions. Thus, without a more explicit sampling design to ascertain geographic and environmental effects, the simplest explanation is that the divergence among the northern and southern regions represents neutral divergence.

Among the four southern populations (Crandon and the three Florida Keys), there is little over all genetic divergence: across all 955 SNPs, the F_ST_ values comparing Crandon *versus* the Florida Keys are approximately 0.09 and within the Florida Keys F_ST_ values are 0.01 (Table 5). Yet, similar to allozyme studies, the mainland population (Crandon) can be distinguished from the Florida Keys by phylogenetic, Structure and discrimination analyses (Fig 2). That is, while most SNPs have little divergence among southern populations, there is a sufficient number of divergent SNPs allowing one to distinguish among the Florida Keys *versus* Crandon (Fig 5). This is most readily seen in the number of SNPs with significant F_ST_ values (44 shared in both analyses or 14 with Bonferroni’s correction). These 44 SNPs have an average F_ST_ value of 0.22. Surprisingly 14 of these SNPs are significant outliers in both outlier tests (see below).

The genetic divergence among the three Florida Keys populations is small with little support for isolation among them: overall F_ST_ values are very small (0.01), and there is no support for differentiation based on phylogenetic or Structure analyses. However the discrimination analysis does suggest that there is sufficient allelic variation to distinguish NNK from the two other Florida Key sites (BPC and BPN). This can be seen in the 29 SNPs (9 with Bonferonni’s correction) that have significant F_ST_ values (Fig 5). These F_ST_ values cannot be confirmed with the AMOVA model because migration is too high for these analyses. The average F_ST_ value among these 29 SNPs is 0.12. Given the low overall difference among the Florida Keys populations (F_ST_ = 0.01) and the fact that only 3% of SNPs have significant F_ST_ values (29/955), it is surprising that 11 of the SNPs are significant outliers.

The important point about both the significant and outlier F_ST_ values (Fig 5) is that they suggest that populations within southern Florida have large and significant differences in allele frequencies that were unexpected based on previous analyses. The p-values associated with these F_ST_ values (Fig 5), the large number of reads for each SNP and the consistency among statistical approaches indicate that these F_ST_ values are biologically relevant and not technical or statistical artifacts.

### Outlier SNP

Outlier SNPs are SNPs whose F_ST_ values exceed the values expected based on extensive permutation of the data [27, 39]. While several biological processes can create excessively large F_ST_ values, the simplest explanation is often that these SNPs are evolving by natural selection [39, 40, 55, 56]. However, FDist2 analyses used in these analyses are associated with false positives [40, 57, 58]. This is because the FDist2 analyses assume an island model of migration that is not always likely to be correct. When this demographic model matches the actual patterns of migrant exchange, FDist2 analyses often perform well with few if any false positives [40]. While an island model may be suitable, we contrast FDist2 with a hierarchical model. While many of the outliers were corroborated with both approaches, especially within south Florida, these results differ in the comparison between the northern and southern Florida populations, most likely due to complex demography associated with the one isolated northern population.

For the FDist2 comparison among southern populations (Crandon *vs*. Florida Keys), an island model seems reasonable. For the Crandon *versus* the Keys, there are 23 FDist2 outliers (average F_ST_ value of 0.26, with the range of p-values from <10^−6^ to 2*10^−4^; Fig 4). The average difference in allele frequencies is 0.353 (compared to 0.156 for SNPs with non-significant F_ST_ values). Most of these outliers (61%) were also found in the hierarchical analysis (Fig 5). These low F_ST_ values, changes in allele frequencies and support from two outlier methods indicate a much stronger divergence for outlier SNPs than other SNPs. Simulation studies suggest that when FDist2 examines populations where the island model is a good approximation, the false positive rate is nearly zero and the true positive rate is approximately 20%. Thus, the number of outliers for the comparison of southern populations is likely to be a good approximation. We therefore conclude that even though southern populations are well connected (F_ST_ values ~0.09), natural selection has affected allele frequencies among these populations. The identification of potentially adaptive divergence among southern populations is where GBS SNP studies differ from previous studies. These results are possible because GBS examines many more loci and thus is more likely to find a locus with significant changes and statistically, one can distinguish loci with large significant F_ST_ values from neutral background by permutation of the data.

The outliers within the Florida Keys are more difficult to model. The exceedingly small F_ST_ values based on all 955 SNPs (~0.01) suggest a near panmictic population. Using FDist2 (island model) there are 11 outlier SNPs (average F_ST_ value of 0.10, range 0.23 to 0.02, range of p-values from <10^−6^ to 2*10^−4^). The average difference in allele frequencies for these 11 SNPs is 0.191 (compared to 0.0095 for SNPs with non-significant F_ST_ values). These 11 SNPs have approximately 100-fold larger F_ST_ values and 20-fold greater changes in allele frequencies than the other SNPs. We could not apply a hierarchical outlier test within the Keys because migration rates were too high for these analyses. Thus, although the differences in allele frequency and extreme p-values (Fig 4) support the outlier test, caution would suggest that not all of these values are true positives. If one assumes these populations are panmictic, then the nine F_ST_ values that exceed Bonferroni’s correction may be indicative of adaptive divergence. What these data suggest is that even among populations that are separated by < 10s of kilometers and with little overall genetic divergence, there are some SNPs affected by natural selection.

We find outlier loci with extremely small p-values (Fig 4) that are corroborated using different approaches (Fig 5). The support for these are especially strong for the population in southern Florida (Crandon and the Keys). Yet, estimations of migration rates (Fig 3) are large (6 individuals per generation) to extremely large (>40 individuals per generation). If some or many of the outlier SNPs are adaptive, this suggests that selection coefficients are large enough to overcome these migration rates.

### Conservation Genomics

Conservation practices need to consider the genetic variation within and among populations [5, 11–13]. Understanding variation within a population provides information on whether there is adequate genetic variation for selection to act on and thus whether a species may adapt to changing environments. Differences among populations provide information on whether there is genetic diversity among habitats. At a minimum, such diversity can indicate whether we need to maintain the connectivity among populations. Connectivity among populations functionally increases effective population size through migration and thus acts to maintain genetic diversity [59, 60]. In the extreme, diversity among populations might also indicate adaptive divergence, in which case individuals from different populations are not fully exchangeable and local extinctions cannot be mitigated by immigration from other locations.

Understanding the genetic diversity within and among populations is more important with GCC because the stress of climate change can cause species extinction, reduce habitat quality, or alter species distributions [1, 2, 61, 62]. With sufficient genetic variation it is possible that species can adapt to GCC, mitigating its effects [14]. With GCC and rising sea levels many coastal habitats will be affected. The questions we are asking are whether there is sufficient variation among isolated estuarine fish within populations and whether there are significant divergences among populations.

These data indicate that within southern Florida *P*. *latipinna* populations there is a high level of genetic variation within all surveyed populations. The southern populations have high migration rates (Fig 3) and no fixed differences, suggesting that conservation of several sites with many individuals would preserve genetic variation best. Second, although it is difficult to predict which loci are important for future adaptive divergence necessary to mitigate GCC effects, the data also show that *P*. *latipinna* displays some adaptive divergence even among geographically close populations. This adaptive divergence found in our genomic data among geographically close populations supports conclusions drawn from previous analyses of life history traits [44, 46]. Although our genomic data does not elucidate the genes involved in adaptive divergence in life history phenotypes, together these studies suggest that local populations readily adapt to their immediate environments. Optimistically, the adaption seen in these populations implies that there may be hope for this species to mitigate GCC effects by evolving adaptive changes.

Finally, we would like to remark on the similarity between our GBS SNP data and previous analyses of similar *P*. *latipinna* populations using allozymes or microsatellites [20, 21]. Nearly all of our conclusions about the genetic variation within and among populations using GBS SNPs are similar to the conclusions from these older technologies. The two exceptions are the number of loci and the ability to use these high numbers of loci in permutation tests to find outlier SNPs (SNPs with excessive F_ST_ values). The large number of loci with small F_ST_ values strongly influences the larger estimates of Nm (effective population size times migration). Additionally, since nearly all of these loci appear to be neutral, we can more accurately detect loci with exceptionally high F_ST_ values. Thus, GBS SNP analyses appear to be similar to previously utilized techniques for determining population structure but provide a substantial improvement on those methods for detecting the potential signature of natural selection.
